# Clinical and Radiographic Features of Cryptococcal Neoformans Meningitis-associated Immune Reconstitution Inflammatory Syndrome

**DOI:** 10.1038/s41598-020-67031-4

**Published:** 2020-06-19

**Authors:** Gang Wu, Xiumei Guo, Yan Wang, Zhijian Hu

**Affiliations:** 1 Professor, Department of Neurology, The First Affiliated Hospital of Fujian Medical University, Fuzhou, Fujian, P.R. China; 2 Department of Neurology, Fujian Provincial Geriatric Hospital; Department of Neurology, North Hospital of Fujian Provincial Hospital, Fuzhou, Fujian, P.R. China; 3 Department of Neurology, First Hospital of Quanzhou, Fujian Province, Quanzhou, Fujian, P.R. China; 4 Department of Epidemiology and Statistics, School of Public Health, Fujian Medical University, Fuzhou, Fujian, P.R. China

**Keywords:** Fungal infection, Central nervous system infections

## Abstract

Cryptococcal meningitis is the most common intracranial infectious fungal disease. After a period of antifungal treatment, as the number of cells in the cerebrospinal fluid decreases, the biochemical indexes improve and the number of cryptococcus reduces, the patient’s condition suddenly worsen. Most of the symptoms are severe headache, raised intracranial pressure, together with impaired clinical nerve function. These presentations are often mistaken for a failure of antifungal treatment. In fact it’s an encephalitis syndrome which is unrecognized by most clinicians: Immune reconstitution inflammatory syndrome (IRIS). To increase awareness we retrospectively analyzed clinical data of 100 cases of cryptococcal neoformans meningitis, among which 26 patients develop CM-IRIS. All patients have been divided into three groups: Group 1, patients who were not treated with glucocorticoid and didn’t experienced IRIS; Group 2, patients who were not treated with glucocorticoid although developed CM-IRIS; Group 3, patients started treatment with glucocorticoid for two weeks with new onset CM-IRIS. Compared with the group treated with glucocorticoid, treatment without glucocorticoid was subjected to a higher risk of incident IRIS. The difference was statistically significant (P < 0.05). Imaging findings demonstrated diseased area of the white matter area, and it looked like commonly in the supratentorial region. Moreover, if it appears in the infratentorial region then must be combined with supratentorial region.

## Introduction

### Objective

Immune reconstitution inflammatory syndrome (IRIS) describes an abnormal modulation that occurs in the recovery of Immune function in immunocompromised patients. This definition elicits in the process of treating HIV-infected patients and was first proposed by DeSimone J.A.*et al*. in 2000^1^. Since then, it’s been widely cited^2^. IRIS can not only be appeared in HIV-infected patients^3.4^, but also be seen in the treatment of non-HIV infected patients with cryptococcal meningitis^5.6^. We have noticed that cryptococcal meningitis (CM), which is a globally invasive fungal disease with a high morbidity and mortality rate, is often concurrent with IRIS, namely cryptococcal neoformans meningitis-associated immune reconstitution inflammatory syndrome (CM-IRIS). CM-IRIS is generally under-diagnosed, which resulted in delayed treatment. Accurate and timely diagnosis is essential and glucocorticoid is found effective^7^ to IRIS. To explore the clinical diagnosis and treatment, we conducted a retrospective study of 100 patients diagnosed as cryptococcal neoformans meningitis, finding that a significant number of these patients developed immune reconstitution encephalitis syndrome.

## Materials and Methods

### Clinical data

From December 2001 to December 2013 we enrolled 193 patients presented with cryptococcal meningitis and admitted to the Department of Neurology, the First Affiliated Hospital of Fujian Medical University. There were 118 men aged 45.41 ± 14.36, 75 women for ages 41.87 ± 16.09. The cases collected in this study have been approved by the Medical Ethics Committee of the First Affiliated Hospital of Fujian Medical University. And all cases met the diagnosis of cryptococcal meningitis by Azure T. Makadzange, *et al*.^8^ The informed consent was obtained from all subjects and their family.

#### Inclusion criteria, exclusion criteria, and grouping of cases of cryptococcal meningitis in this study

**Figure 1:**
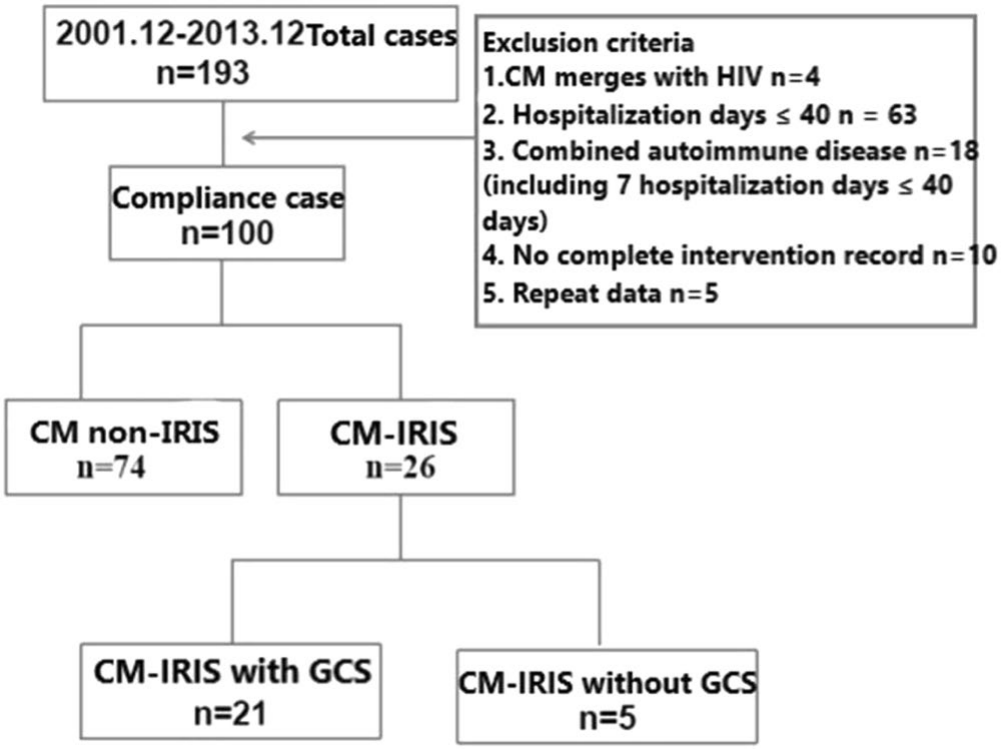
We enrolled 193 patients presented with cryptococcal meningitis from December 2001 to December 2013, 100 patients met the requirements according to inclusion criteria and were divided into two groups (CM-IRIS group and CM non-IRIS group) depends on whether IRIS developed or not and conducted comparative analysis of curative effectiveness. Furthermore we divided CM-IRIS group into two groups: CM-IRIS treated with GCs and CM-IRIS treated without GCs, and did comparative analysis of not only curative effectiveness but also incidence of IRIS.

initially 193 patients were enrolled based on the inclusion criteria and exclusion criteria as follows: Inclusion criteria: 1. Hospital stay≧40 days; 2. At least twice (upon admission and discharge) cranial MRI scan; 3. A complete record of therapeutic interventions that serum and cerebrospinal fluid are recorded every fortnight. Exclusion criteria: 1. Cryptococcal meningitis in HIV-Infected patients (4 cases); 2. Length of stay≦40 days (63 cases); 3. Combined with autoimmune diseases, such as systemic lupus erythematosus (SLE), erythrodermic psoriasis (18 cases); 4. No complete record of intervention (10 cases); 5. Duplicated data (5 cases). According to all the above-mentioned inclusion and exclusion criteria, 100 patients met these requirements and were included in the final analysis. There were 59 males aged 44.17 ± 12.69 and 41 females aged 42.10 ± 15.88, as shown in Fig. 1.

#### Diagnostic criteria for non-HIV IRIS

**Figure 2:**
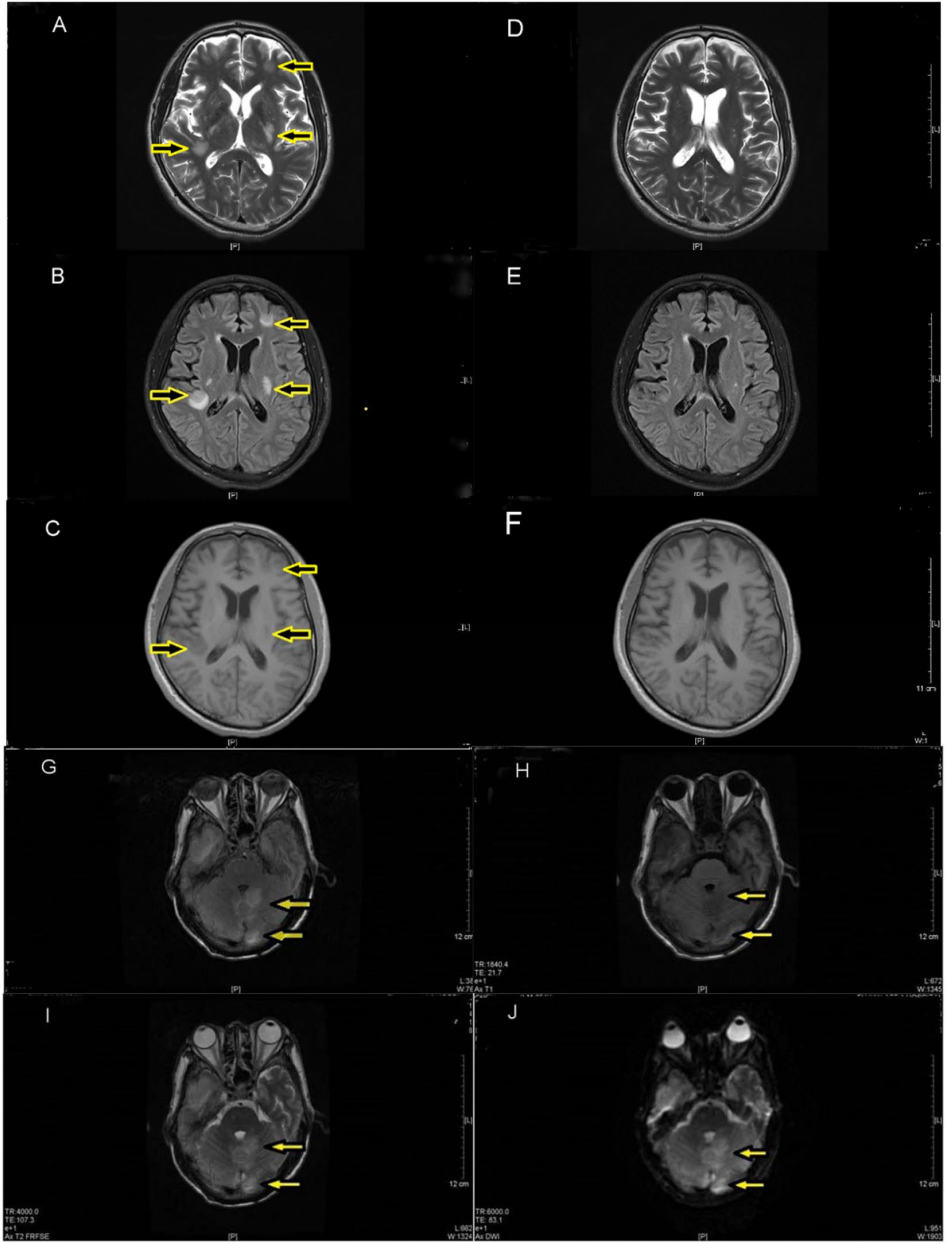
Supratentorial lesions as showed in picture A, B, C, D; Picture A, B, C are T1W1 imaging, T2W1 and Flair imaging respectively of a patient diagnosed with CM before being treated with GCs, 43th days after being admitted to hospital. T1W1 displayed low signal while T2W2 and Flair imaging displayed high signal which distributed beneath the left frontal cortex, within the white matter of basal ganglia and the right cistern (as indicated by arrows). Picture D, E, F are the same T1W1 imaging, T2W2 and Flair imaging of the patient after being treated with GCs for 21 days. No significant abnormality was seen in T1W1 and T2W2 imaging while the high signal in the left frontal cortex and the white matter of right cistern showed by Flair was obviously decreased or even disappeared in comparison to the previous (relatively compare with the area indicated by arrows in Picture A, B, C). Infratentorial lesions showed in picture G, H, I, J by Flair, T1W1, T1W2 and DWI imaging, the yellow arrow represents inflammatory signal. In this figure they weren’t the same person for pictures of supratentorial and infratentorial lesions.

The diagnosis of CM-IRIS in this study is in compliance with the diagnostic criteria of non-HIV IRIS proposed by Nina Singh *et al*. 2007^9^. Of all the included patients in the study, 26 met the diagnostic criteria for HIV-negative CM-IRIS, including 15 men aged 49.93 ± 9.47 years old and 11 women aged 43.91 ± 20.35. 74 patients reported non-IRIS cryptococcal meningitis (CM without IRIS), including 44 males (aged 42.20 ± 13.14) and 30 females (aged 41.43 ± 14.26). All cases were detected by MRI on FLAIR imaging, T2WI imaging and T1WI imaging to check whether abnormal inflammatory signal exists or not. The inflammatory signal obviously decreased or even disappeared after treating with Glucocorticoid (GCs), as shown in Fig. 2 (Fig. 2. A-F). Different inflammatory area divides into 3 types: supratentorial and infratentorial lesions (Fig. 2. G-J) and mixed type. Mixed type means both supratentorial and infratentorial regions were affected, and most of the situations is white matter lesions. Once infratentorial white matter damage developed, it must be combined with supratentorial white matter lesions. Among the 26 cases, 20 cases (76%) are supratentorial lesions and 6 belong to mixed type, no pure infratentorial white matter lesions observed.

### Methods

#### Laboratory data sources

Data including cerebrospinal fluid biomarkers, biochemical and cytological results was based on the clinical examination data from the clinical laboratory of the First Affiliated Hospital of Fujian Medical University and the Center for Cerebrospinal Fluid Examination of Fujian Province. The imaging data was provided by the Department of Radiology, the First Affiliated Hospital of Fujian Medical University. GE’s SIGNA EXCITE 1.5 T Twin Speed and SIEMENS’s 3.0 T MEGNETOM Verio were adapted as magnetic resonance imaging (MRI) scanner during the study.

#### Research design

We performed a retrospective study. First of all, we compared the baseline data of these subgroups and checked if any difference exists on gender ratio, ages and length of stay: CM-IRIS and CM non-IRIS, CM-IRIS treated with GCs and without GCs. Besides, 100 cases applied not only to the including criteria but also to the excluding criteria were collected to do statistical induction on clinical symptoms, clinical signs and cerebrospinal fluid components. Secondly, we divide antifungal drugs into three comparison combinations (A, B and C) to examine their therapeutic effects to CM-IRIS. Then we replicated our analysis according to whether the patient developed IRIS and treated with GCs. Dynamic observation was performed in patients diagnosed of CM-IRIS and CM non-IRIS by recording manifestations, cerebrospinal fluid pressure, biochemical indexes and MR imaging changes. Notice the time at onset for CM-IRIS in the whole course of cryptococcal meningitis. Track down the indexes changes in different stages of CM-IRIS in order to do comparison and evaluate whether GCS is effective. Finally, research the comparison results of CM-IRIS and CM non-IRIS, CM-IRIS treated with GCs and without GCs and figure out the incidence of IRIS.

#### Statistical methods

SPSS19.0 statistical data package was used to analyze the data, which includes number, average age, average length of stay, rate of the improved and the incidence of disease. Algorithm data adopted mean value plus or minus standard deviation to perform, and measurement data adopted percentage. Gender ratio of CM-IRIS and CM non-IRIS patients was evaluated by Chi-square test with four-fold table; Average age and average length of stay of CM-IRIS and CM non-IRIS, CM-IRIS treated with GCs and CM-IRIS without GCs applied T-test to do statistical comparison. The clinical biochemical indicators of CM-IRIS patients which was divided into 3 comparison groups according to three different stage were compared by means of single-factor ANOVA. It turns out that P value of ANOVA is significant so that we continued to do pairwise comparison.

## Results

### **The baseline characteristics of patients with CM and developed with IRIS or not, and patients confirmed with CM-IRIS and treated with GCs or not**

CM-IRIS: there is a total of 26 cases, including 15 males and 11 females, with a mean age of 47.38 ± 15.00 years and an average hospital stay of 130.77 ± 88.46 days. CM non-IRIS group: there are 74 patients of which 44 are men and 30 are women, with a mean age of 41.89 ± 13.51 years and an average hospital stay of 121.09 ± 70.74 days. While CM-IRIS treated with GCs is 21 cases (14 males and 7 females) with a mean age of 45.43 ± 14.89 years and an average hospital stay of 146.57 ± 89.70 days. CM-IRIS treated without GCs is 5 cases (2 males and 3 females) with a mean age of 55.60 ± 13.90 years (38 to 88 years old) and an average hospital stay of 64.00 ± 43.09 days. There have been no significant differences in gender, age or length of hospital stay between the CM-IRIS group and the CM non-IRIS group (P-values are more than 0.05). Also, no significant differences in gender, age or length of hospital stay between the group of CM-IRIS treated with GCs and the group treated without GCs (P-values are more than 0.05), as shown in Table 1.

**Table 1:** The baseline characteristics of patients with CM and developed with IRIS or not, and patients confirmed with CM-IRIS and treated with GCs or not.

Group	CM-IRIS (n = 26)	CM non-IRIS (n = 74)	CM-IRIS with GCs (n = 21)	CM-IRIS (without GCs) (n = 5)	P_1_ P_2_
Project					
Male to female ratio	15:11	44:30	14:7	2:3	0.875 0.340
Median age	47.38 ± 15.00	41.89 ± 13.51	45.43 ± 14.89	55.603 ± 13.90	0.086 0.178
Average hospital stay	130.77 ± 88.46	121.09 ± 70.74	146.57 ± 89.70	64.00 ± 43.08*	0.576 0.060

* Note: For economical reason 4 of the 5 patients in the group of CM-IRIS treated without GCs were discharged from the hospital and transferred to local hospital for further treatment, thus the average hospital stay was short. P1 was the P value for the data of CM-IRIS and the CM non-IRIS group; P2 was the P value for the data of the two groups that CM-IRIS with GCS treatment and without. Both P1 and P2 were above 0.05, hence the data of gender, age and length of hospital stay didn’t reach statistical significance.

### Different combinations of antifungal treatments have been shown to have no effects on not only the treatment of CM but also incident CM-IRIS and CM non-IRIS

In this study we identified the methods for evaluation of effective treatments to people with CM, that is, clinical symptoms and cerebrospinal fluid biomarkers (Supplementary data, Table 1). Three combinations of antifungal treatments in this study are as follows: Group A: Voriconazole/Fluconazole+5-Fluorocytosine; Group B: Amphotericin B + 5-Fluorocytosine; Group C: Voriconazole/Fluconazole+Amphotericin+5-Fluorocytosine. We adopted χ^2^ which is a table contains a matrix of rows and columns to conduct the examination: χ^2^ = 11.802, P = 0.299. Statistical results suggested that different combinations of antifungal treatments had been shown to have no effects on treatment to CM; Through adopting χ^2^ (χ2 = 0.696, P = 0.706) to do the similar examination: we found the same results in the treatment of different antifungal drugs to CM-IRIS and CM-no IRIS (Supplementary data, Table 2).

**Table 2:** To compare clinical biochemical indicators of the three periods of 26 cases with CM-IRIS.

sample project	Group 1 (n = 26)	Group 2 (n = 17)	Group 3 (n = 12)	F value	P value	
Cerebrospinal fluid	pressure	198. 46 ± 68.74*	269.12 ± 68.19*^▲^	180.41 ± 81.64^▲^	6.958	0.002
	glucose	3.06 ± 1.42	2.27 ± 1.26	2.73 ± 1.40	1.723	0.189
	protein	1.35 ± 0.91*	0.82 ± 0.70*	0.86 ± 0.50	5.344	0.008
	chloride	120.15 ± 7.47	116.12 ± 7.54	117.75 ± 5.61	1.703	0.192
	leukocyte	140.31 ± 146.96*^▲^	59.94 ± 75.50z*	48.83 ± 39.15^▲^	4.005	0.024
	Lymphocyte %	72.65 ± 11.52*^▲^	80.59 ± 7.18*	82.42 ± 4.44^▲^	6.320	0.003
serum	leukocyte	9.69 ± 3.84	8.96 ± 3.47	9.23 ± 2.65	0.235	0.792
	Lymphocyte	1.42 ± 0.58	1.56 ± 0.68	1.76 ± 0.73	1.158	0.322

Note: Grouping: Group 1: haven’t developed IRIS yet and not treated with GCs; Group 2: have developed IRIS and not treated with GCs; Group 3: have developed IRIS and treated with GCs for two weeks. Statistical methods: Firstly, we adopted analysis of variance (statistical analysis) to do the analysis, the result indicated that there were significantly different changes of intracranial pressure of cerebrospinal fluid, protein, the leucocytes and lymphocyte percentage of the three groups, P values were all less 0.05. Subsequently we made comparison between each group, asterisks (*) and triangles (▲) means statistically significant, of which the P values were all less than 0.05.

### **To compare three stages of CM-IRIS according to the progression of disease and whether treated with GCs or not**

**Figure 3:**
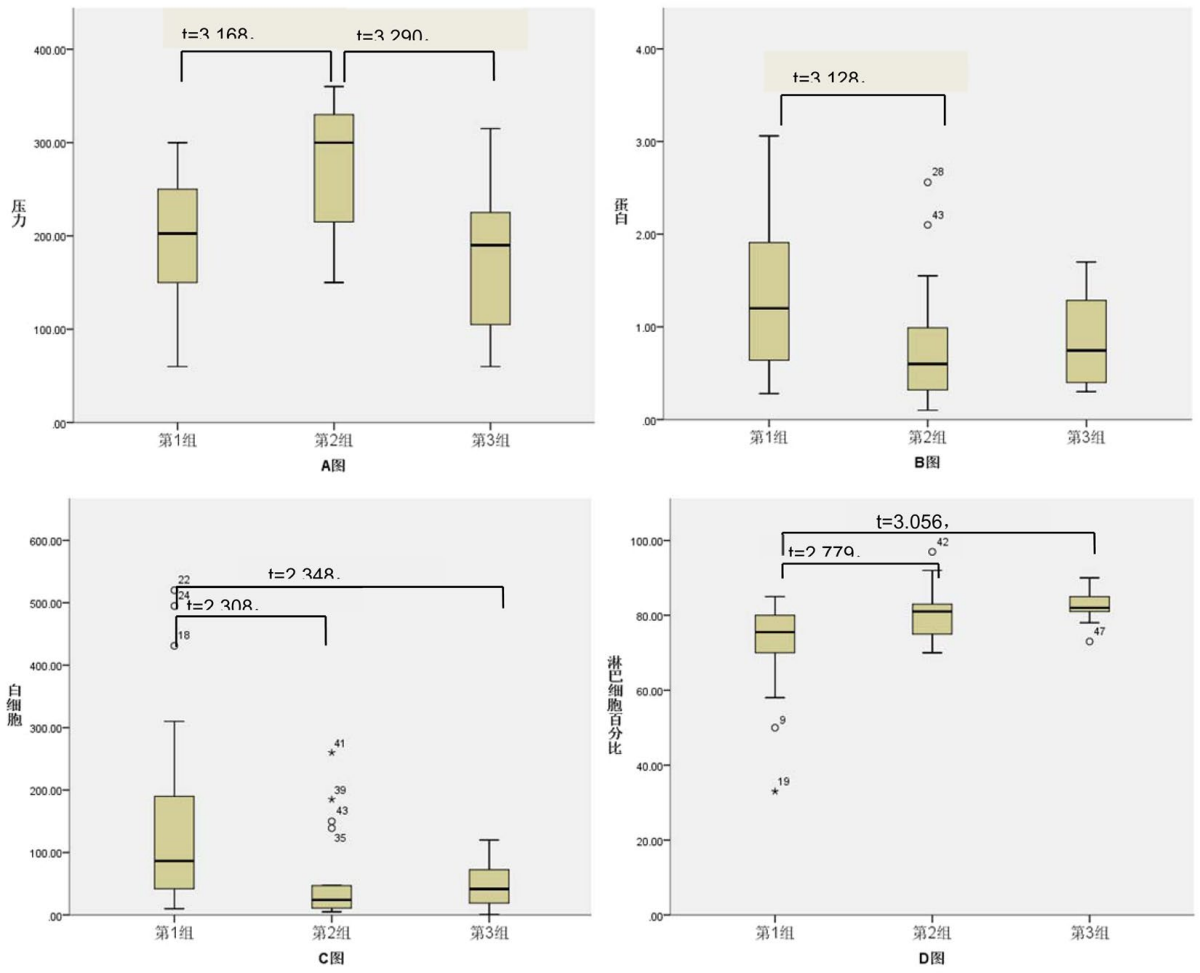
Note: I. Grouping and illustration: Group 1: haven’t developed IRIS yet and not treated with GCs; Group 2: have developed IRIS and not treated with GCs; Group 3: have developed IRIS and treated with GCs for two weeks. Picture A, B, C, D previously shown defines the intracranial pressure, protein, the leucocytes and lymphocyte percentage of cerebrospinal fluid. II. Statistical methods: The results of variance analysis indicated that difference of changes among the intracranial pressure, protein, the leucocytes and lymphocyte percentage of cerebrospinal fluid were statistically significant, F values were 6.958, 5.344, 0.008, 4.005, 6.320 respectively, P values were all less than 0.05. The comparison between each group was checked by LSD-t test, and the results are shown in the above figures.

In this study 26 patients confirmed with CM-IRIS were divided into three groups: Group 1: haven’t developed IRIS yet and not treated with GCs; Group 2: have developed IRIS and not treated with GCs; Group 3: have developed IRIS and treated with GCs for two weeks. We made comparison on the changes of intracranial pressure of cerebrospinal fluid, glucose, protein, chloride, the leucocytes and lymphocyte percentage. Firstly, we adopted analysis of variance (statistical analysis) to do the analysis, the result indicated that there were significantly different changes of intracranial pressure of cerebrospinal fluid, glucose, protein, chloride, the leucocytes and lymphocyte percentage of the three groups (F values 6.958, 5.344, 0.008, 4.005 and 6.320 respectively, P values 0.002, 0.008, 0.024, 0.003 respectively). On the basis of analysis of variance, we adopted LSD-t test to do data comparison among groups, the results showed intracranial pressure of cerebrospinal fluid of CM-IRIS is obviously higher than CM non-IRIS group (t = 3.168, p < 0.05), also obviously higher than CM-IRIS treated with GCs (t = 3.290, p < 0.05), the difference is significant. With regard to protein of cerebrospinal fluid, the leucocytes and lymphocyte percentage, the protein of CM IRIS group treated without GCs was obviously less than CM non-IRIS group (t = 3.128, p < 0.05), as well as leucocyte count, that was to say Group 1 was higher than Group 2 (t = 2.308, p < 0.05), more than Group 3 (t = 2.348, p < 0.05) too, the difference is of overt significance. As for lymphocyte percentage, compared with the CM-IRIS group, CM non-IRIS (haven’t developed IRIS yet) group was higher, that’s, Group 2 is higher than Group 1 (t = 2.779, p < 0.05), furthermore Group 3 is higher than Group 1 too (t = 3.056, p < 0.05), the difference was significant. Above-mentioned results can be found in Table 2, Fig. 3.

### To explore the impact whether CM patients treated with or without GCs have on the incidence of CM-IRIS

In this study both CM-IRIS and CM non-IRIS were divided into Group A and Group B according to whether treated with or without GCs. Group A’s incidence of IRIS was higher than Group B’s (χ^2^ = 6.021, P = 0.014), the difference was significant. As shown in Table 3.

**Table 3:** To explore the impact if CM patients treated with or without GCs have on the incidence of CM-IRIS.

Group	CM-IRIS	CM non-IRIS	Total	Incidence (%) of CM-IRIS
A	5	3	8	62.50
B	21	71	92	22.83
Total	26	74	100	26

Note: Group A: treated without GCs; Group B: treated with GCs. Adopted χ 2 test which contained a matrix of rows and columns to check the formula: χ^2^ = 6.021, P = 0.014.

### The timing of onset CM-IRIS throughout the whole course of CM

We examined the timing of new onset IRIS of all 21 patients with CM-IRIS.

Since the onset of CM, which generally begins with symptoms of headache, fever and other known clinical symptoms, following antifungal treatment, then CM-IRIS manifests and eventually patients have been diagnosed with IRIS, the length of time is 47.5 ± 28.44 days. Of which 1 case is no more than 20 days, 11 cases are about 20 to 40 days, 8 cases are 40 to 60 days, 3 cases are 60 to 80 days, 2 cases are 100 to 120 cases, one is 120 to 140 days. In most cases the time span is around 20 to 60 days.

and J maps FLAIR, T1WI, T2WI, and DWI, and the yellow arrow marks showed inflammatory signals The white matter lesions under the curtain and in the supratentorial were from different patients in this images data.

## Discussion

Cryptococcal meningitis is the most common intracranial infectious fungal disease, usually associated with immunocompromised patients. After active antifungal treatment, some patients are cured, and a considerable number of patients still fail to treat^10^. After a period of antifungal treatment, as the number of cells in the cerebrospinal fluid decreases, the biochemical indexes improved and the number of cryptococcus reduces, the patient’s condition suddenly worsen. Most of the symptoms are severe headache, raised intracranial pressure, together with impaired clinical nerve function. These presentations are often mistaken for a failure of antifungal treatment. In fact it’s an encephalitis syndrome which is unrecognized by most clinicians: Immune reconstitution inflammatory syndrome (IRIS). Available data indicate that up to 20% of patients with complication of cryptococcal meningitis (CM) die from concurrent immune reconstitution encephalitis syndrome^11^.

In addition to improving the clinical symptoms, we found that GCs has a special effect on this disease in this study. As shown in Table 3, compared with Group 3 which didn’t treated with GCs, the intracranial pressure of cerebrospinal fluid of Group 2 that treated with GCs was obviously reduced, as well as clinical symptoms got better. P values were all less than 0.05 therefore the difference was significant.

Data from this study showed that when IRIS occurred, clinical characteristic changed and started with growing headache, increased pressure of cerebrospinal fluid, protein and leucocytes decreased but lymphocyte percentage rose. After using GCs headache was immediately relieved. Two weeks later only to find the pressure of the cerebrospinal fluid obviously went down, no significant changes for other ingredients. No matter treated with GCs or not the serum inflammatory indexes of CM-IRIS didn’t reach statistically significance, which was consistent with the views of Chang C. C *et al*.^12^.

CM can also experience with immune reconstitution inflammatory syndrome (CM-IRIS) in an early stage^5^. Due to the lack of characteristic manifestations of cerebrospinal fluid, it is more likely to be misdiagnosed. Therefore, the brain IRIS occurs at this time, and the imaging basis occupies a very important position^13^. The cases collected in this study are not early cases therefore they’re not difficult to identify clinically.

The average time of brain IRIS occurred in this study was 47.50 ± 28.44 days, and the predilection time span was 20–60 days. The literature reports that brain IRIS of cryptococcal meningitis often occurs around 6 weeks after antifungal treatment^14,15^ Premature use of GCS in CM antifungal therapy may affect antifungal therapy^16^, Therefore, based on the average time of brain IRIS occurred in this group and the time of brain IRIS reported in the literature reports, it may be reasonable to recommend the addition of GCs for about 6 weeks during antifungal therapy for CM.

In addition, according to the imaging results CM-IRIS’s imaging abnormalities occurred in the brain parenchyma on the cerebellum, pure supratentorial lesions were more common, pure infratentorial lesions hadn’t been observed. This study shows that once the lesions appeared under the curtain, it must be combined with the supratentorial lesions.

In summary, through analyzing the clinical symptoms, routine cerebrospinal fluid biomarkers and imaging changes, CM-IRIS of the brain can be correctly identified. Simultaneously, correct antifungal therapy combined with timely GCs treatment can significantly alleviate or reduce the occurrence of brain IRIS.

## Supplementary information

Supplementary information.
