# Supplementary material for: Clinical and Radiographic Features of Cryptococcal Neoformans Meningitis-associated Immune Reconstitution Inflammatory Syndrome
Source: Sci Rep. 2020 Jun 19;10:9948. doi: 10.1038/s41598-020-67031-4 (PMC7305181; doi:10.1038/s41598-020-67031-4)
Supplement: Supplementary file 1 — Supplementary information. [file 41598_2020_67031_MOESM1_ESM.pdf]

Clinical and Radiographic Features of Cryptococcus Neoformans Meningitis-associated Immune Reconstitution Inflammatory syndrome

[1]Gang Wu*, [2]Xiumei Guo*， [3]Yan Wang， [4]Zhijian Hu

Corresponding author:
Correspondence to Gang Wu, gangwu57@hotmail.com

Author affiliation
1. Department of Neurology, The First Affiliated Hospital of Fujian Medical University, Fuzhou, Fujian, P. R. China
2. Department of Neurology, Fujian Provincial Geriatric Hospital; Department of Neurology, North Hospital of Fujian Provincial Hospital, Fuzhou, Fujian, P.R.China
3. Department of Neurology, First Hospital of Quanzhou, Fujian Province, Quanzhou, Fujian, P.R.China
4. Department of Epidemiology and Statistics, School of Public Health, Fujian Medical University, Fuzhou, Fujian, P.R.China

* Equal contributors

Supplementary Data：

Suppl Table 1. To identify the methods for evaluation of effective treatments to people with CM, that is, clinical symptoms and cerebrospinal fluid biomarkers.

Prognosis	1. Healed	2. Significant effect	3. Effective	4. Invalid	5. Death or worsening discharge
Clinical manifest a tion	Clinical symptoms and signs completely disappeared without complications;<br><br>Cerebrospinal fluid examination ≥ 2 times normal, ink smear negative ≥ 3 times normal; No obvious abnormalities were observed in MRI;<br><br>The last cerebrospinal fluid examination pressure: 80-180mmH20;<br><br>Total protein: 0.15-0.45g/L;<br><br>Glucose: 2.5-4.5mmol/L;<br><br>Chloride: 120-132 mmol/L;<br><br>White blood cell	Clinical symptoms and signs were significantly improved with or without complications;<br><br>The last cerebrospinal fluid examination was negative for the smear of the ink, which was obviously improved, but did not reach normal:<br><br>Cerebrospinal fluid pressure: 180-200mmH20;<br><br>Total protein: 0.45-0.60g / L;<br><br>Glucose: 2.20-2.50mmol/L;<br><br>Chloride: 110-120mmol/L;<br><br>White blood cell count: 8-20 x 10^{6} / L	Clinical symptoms and signs improved slightly with or without complications;<br><br>The last cerebrospinal fluid examination was slightly improved, the ink smear was positive, but the number of cryptococci was significantly reduced compared with the previous one;<br><br>Cerebrospinal fluid pressure: 200-250mmH20<br><br>Total protein: 0.60-2.00g / L;<br><br>Glucose: 1.5-2.2mmol/L;<br><br>Chloride: 100-109mmol/L;<br><br>White blood cell count: 20-60 × 10^{6} / L	After treatment, the clinical symptoms and signs have not improved or aggravated, and obvious complications.<br><br>The last cerebrospinal fluid examination did not improve. The ink smear was positive and the number of cryptococci was not reduced.<br><br>Cerebrospinal fluid pressure: >250mmH20;<br><br>Total protein: >2g/L;<br><br>Glucose: <1.5mmol/L;<br><br>Chloride: <100mmol/L;<br><br>White blood cell count: >60×106/L.	death deterioration

count: 0-8 x 10^6
/ .

Suppl Table 2. Different combinations of antifungal treatments have been shown to have no effects on not only the treatments of CM but also incident CM-IRIS and CM non-IRIS.

Efficacy	A	B	C
Healed	9.09%	0	3.57%
Significant effect	36.36%	18.18%	37.50%
Effective	45.45%	81.82%	42.86%
Invalid	0	0	3.57%
Death	0	0	7.14%
worsening discharge	9.09%	0	5.36%
CM-IRIS	24.24%	36.36%	25.00%
CM non-IRIS	75.76%	63.64%	75.00%
Total	33	11	56

Note: Group A: Voriconazole/Fluconazole+5-Fluorocytosine; Group B: Amphotericin B+5-Fluorocytosine; Group C: Voriconazole/Fluconazole+Amphotericin+5-Fluorocytosine.
We adopted χ^2 which is a table contains a matrix of rows and columns to conduct the examination: χ^2=11.802, P=0.299.
Statistical results suggested that different combinations of antifungal treatments had been shown to have no effects on treatment to CM; Through adopting χ^2 (χ2=0.696， P=0.706) to do the similar examination: we found the same results in the treatment of different antifungal drugs to CM-IRIS and CM-no IRIS.
